# Investigating Individuals’ Preferences in Determining the Functions of Smartphone Apps for Fighting Pandemics: Best-Worst Scaling Survey Study

**DOI:** 10.2196/48308

**Published:** 2023-08-15

**Authors:** Richard Huan Xu, Lushaobo Shi, Zengping Shi, Ting Li, Dong Wang

**Affiliations:** 1 Department of Rehabilitation Sciences Hong Kong Polytechnic University Kowloon Hong Kong; 2 School of Health Management Southern Medical University Guangzhou China

**Keywords:** best-worst scaling, BWS, smartphone app, app, pandemic, preference, health care, survey

## Abstract

**Background:**

Smartphone apps have been beneficial in controlling and preventing the COVID-19 pandemic. However, there is a gap in research surrounding the importance of smartphone app functions from a user’s perspective. Although the insights and opinions of different stakeholders, such as policymakers and medical professionals, can influence the success of a public health policy, any strategy will face difficulty in achieving the expected effect if it is not based on a method that users can accept.

**Objective:**

This study aimed to assess the importance of a hypothetical smartphone app’s functions for managing health during a pandemic based on the perspective of user preferences.

**Methods:**

A cross-sectional and web-based survey using the best-worst scaling (BWS) method was used to investigate the general population’s preferences for important smartphone app functions. Participants were recruited from a professional surveying company’s web-based surveying panel. The attributes of the BWS questionnaire were developed based on a robust process, including literature review, interviews, and expert discussion. A balanced incomplete block design was used to construct the choice task to ensure the effectiveness of the research design. Count analysis, conditional logit model analysis, and mixed logit analysis were used to estimate preference heterogeneity among respondents.

**Results:**

The responses of 2153 participants were eligible for analysis. Nearly 55% (1192/2153) were female, and the mean age was 31.4 years. Most participants (1765/2153, 81.9%) had completed tertiary or higher education, and approximately 70% (1523/2153) were urban residents. The 3 most vital functions according to their selection were “surveillance and monitoring of infected cases,” “quick self-screening,” and “early detection of infected cases.” The mixed logit regression model identified significant heterogeneity in preferences among respondents, and stratified analysis showed that some heterogeneities varied in respondents by demographics and COVID-19–related characteristics. Participants who preferred to use the app were more likely to assign a high weight to the preventive functions than those who did not prefer to use it. Conversely, participants who showed lower willingness to use the app tended to indicate a higher preference for supportive functions than those who preferred to use it.

**Conclusions:**

This study ranks the importance of smartphone app features that provide health care services during a pandemic based on the general population’s preferences in China. It provides empirical evidence for decision-makers to develop eHealth policies and strategies that address future public health crises from a person-centered care perspective. Continued use of apps and smart investment in digital health can help improve health outcomes and reduce the burden of disease on individuals and communities.

## Introduction

Smartphone apps have proven to be beneficial in controlling and preventing the COVID-19 pandemic. These apps can provide up-to-date information on the pandemic, including exposure notifications and contact tracing, which can facilitate government decision-making. Individuals can also use these apps to track symptoms and monitor their health [1]. However, while smartphone apps have been developed to help fight COVID-19, they may not be effective due to limitations. For example, the required functions may be difficult to find, or some people may not feel comfortable using this technology [2]. Additionally, some apps are not user-friendly, which makes them less accessible to certain populations [3].

Another significant barrier with many apps available is that it can be demanding to choose the most suitable one [4]. Navigating through them can be difficult, as each of them has its own interface and set of features. This can be overwhelming for users, leading to confusion and frustration. Ultimately, it reduces the overall effectiveness of these apps in mitigating the spread of the virus. Even the most effective app only works if enough people use it, so app adoption rates are an important factor [5]. Despite their limitations, smartphone apps can still be a useful tool in managing health. They provide a platform for health authorities to communicate with the public and offer guidance on how to stay safe during pandemics. However, given the finite resources that governments can invest in eHealth or mobile health programs, and the limited capabilities of technology, it is vital for developers to understand users’ preferences before developing app functions. By taking users’ preferences into account, developers can ensure that smartphone apps provide accurate information and useful functions that meet people’s needs for managing their health.

Currently, however, there is a gap in the research surrounding the importance of smartphone app functions from a user’s perspective [2,3]. It is vital to know how heterogeneity varies across different demographics and user preferences, as this could provide valuable insights into successful app development in the future. As telecommunications continue to advance, smartphone apps are believed to play an increasingly important role in fighting future public health crises. To ensure the effectiveness of these apps, it is crucial to involve the perspectives of the public in the development process and prioritize clear communication. Therefore, the objective of this study was to assess the importance of a hypothetical smartphone app’s functions for managing health during a pandemic based on users’ preferences in the general population of China.

## Methods

### Best-Worst Scaling Method

Best-worst scaling (BWS) is a method used to measure individual preferences, and it has been categorized into three types: cases 1, 2, and 3. In case 1, researchers explore the preferences of a population of interest regarding each item of a particular list of objects. Individuals under observation are presented with a set of objects and asked to choose separately the best and the worst object. In case 2, researchers focus on the importance ranking of items presented in a single profile structure developed by combining attributes at various levels. Case 3 presents multiple profiles to the individuals and asks them to choose the best and worst one from each choice set. In this study, we used the BWS case 1 method.

### Development of Attributes of BWS Questionnaire

The development of the BWS questionnaire was a robust and meticulous process that involved several steps. The first step involved drafting 8 possible attributes based on 3 literature reviews [1,6,7]. This developed the foundation of this study and helped the team form a preliminary understanding of the current landscape of mobile health solutions in the pandemic. In the second step, the team further reviewed existing evidence on barriers and facilitators to mobile app use in reducing COVID-19 risks. This helped to identify additional attributes that could be added to the attribute pool. The team sought to ensure that the attributes were comprehensive and covered possible barriers and facilitators to mobile app use [2-5,8-16]. After step 2, a list of 12 attributes was identified. In the third step, the team randomly recruited 10 medical university students and conducted semistructured one-on-one interviews to discuss the feasibility of the attributes. The aim was to identify any potential issues with the attributes and to revise them as necessary. The team combined and revised 6 of the attributes and additionally created a set of 11 attributes. The inclusion of young people’s perspective was important, as they are a key demographic group that could benefit from mobile health solutions in the pandemic. In the fourth step, an expert group consisting of public health researchers, health policymakers, and a mobile app developer reviewed the list of attributes. The panel revised some wording, resulting in a draft of 10 attributes. Their input helped to ensure that the attributes were clear and concise. In the fifth step, a convenience sample of 10 members of the general public was recruited to provide feedback on the 10 attributes from a user perspective. The aim was to ensure that the attributes were relevant to the general public and that they could be easily understood. Although no further revisions were made, the team took their feedback into consideration. Last, the team reviewed all previous information and evidence and included one additional attribute in the list. Finally, a set of 11 attributes (ie, functions) was confirmed to be used to develop the BWS questionnaire. A description of each attribute is provided in Multimedia Appendix 1.

### Experiment Design

To ensure the effectiveness of the research design, a balanced incomplete block design (BIBD) was used to construct the choice task. BIBD is the optimal way to ensure that each attribute level is equally replicated and appears within a block with every other attribute level an equal number of times [17]. This method guarantees that there is an equal probability of selecting each attribute in the questionnaire. The experiment design is displayed in Table 1. In this study, 11 choice task profiles, each with 5 attributes, were created. Participants were asked to select 2 attributes, with one being the most important and the other being the least important function that the app should provide. In this study, each participant had to make a total of 22 choices (11 best and 11 worst).

**Table 1:** The balanced incomplete block design of the best-worst scaling questionnaire. Each choice set contained 5 items, and each item appeared 5 times across the choice sets.

Items	Choice set										
	1	2	3	4	5	6	7	8	9	10	11
1			✓	✓		✓			✓	✓	
2		✓			✓	✓		✓	✓		
3	✓			✓				✓	✓		✓
4	✓		✓		✓			✓		✓	
5					✓		✓		✓	✓	✓
6	✓	✓	✓				✓		✓		
7		✓		✓			✓	✓		✓	
8			✓			✓	✓	✓			✓
9	✓	✓				✓				✓	✓
10	✓			✓	✓	✓	✓				
11		✓	✓	✓	✓						✓

### Data and Participants

Data used in this study were obtained from a web-based cross-sectional survey in January 2023. All participants were recruited from the web-based panel of Wenjuanxing, a survey company. The inclusion criteria were as follows: (1) age ≥18 years; (2) ability to understand and read Chinese; and (3) agreement to provide informed consent. All eligible participants were invited to join to complete a web-based questionnaire. There is no gold standard for determining the sample size for a BWS case 1 study. A recent systematic review of health-related studies found that the sample size for BWS case 1 studies ranged between 15 and 803, with a median of 175 [18]. In addition, according to Louviere et al [19], a sample of 510 is sufficient to meet the required assumption (*P*<.05) based on Thompson’s formula for estimating the sample size for multinomial proportions data. However, a larger sample provides greater power. Therefore, a convenience sample size of 2000 was chosen for this study. The first page of the questionnaire contained an informed consent section that participants needed to read and agree to before proceeding. The questionnaire consisted of 2 sections: the first was the BWS questionnaire and the second was a series of questions asking for respondents’ personal information, such as gender, age, and COVID-19–related information. We ensured data quality by working with the survey company on a series of indicators, including completion time, IP address, and an artificial intelligence formula developed by the company to analyze the participants’ response patterns to avoid random error. The survey was anonymous and did not collect any personally identifiable information. All the BWS questions were predefined as compulsory to ensure no missing data needed to be imputed in our analysis.

### Statistical Analysis

A count analysis was performed to examine the frequency of choice for each BWS attribute. The BWS score was calculated as the difference between the number of times an attribute was selected as the most important and the number of times that attribute was selected as the least important for each of the 11 attributes. A positive BWS score indicated that the attribute was chosen more often as the most important, while a negative score indicated the opposite. Another outcome measure was mean BWS score, which was calculated by dividing the BWS score by the number of participants who responded to each attribute.

The data were further analyzed using 2 regression methods: a conditional logit model (CLM) and a mixed logit (MXL) model. Both methods incorporate the logit procedure, where each attribute is dual-coded. The “best” variable is assigned a value of 1 if the attribute is chosen as the most important, and 0 otherwise. Meanwhile, the “worst” variable is assigned a value of –1 if the attribute is chosen as the least important, and 0 otherwise. These variables are used to estimate propensity scores for the presence of an attribute in a specific combination of attributes.

Unlike CLM estimation, MXL accommodates unobserved taste heterogeneity by specifying preference parameters as random variables with means and SDs rather than fixed parameters. It can derive an SD to indicate unexplained variation around each attribute’s mean. An SD value significantly departing from zero indicates significant heterogeneity. The Akaike information criterion (AIC) and log likelihood (LL) values were used to assess model fit. Smaller AIC values indicate a better fit, while higher LL values indicate a better model. Stratified analysis was used to detect the heterogeneity of mean BWS score across different subgroups (divided by COVID-19–related and socioeconomic characteristics) with the robust Yuen method for trimmed means (bootstrap=1000). R (R Core Team) was used to design the BWS and perform all the statistical analyses.

### Ethical Considerations

The study protocol and informed consent procedure were approved by the institutional review board of the Hong Kong Polytechnic University (HSEARS20221029001). All study data were anonymous. Respondents were provided with a cash incentive (RMB 2; US $0.30) by the survey company to encourage their participation.

## Results

### Complete Cases and Dropouts

A total of 4383 individuals were invited to complete the web-based survey, of whom 2606 agreed to participate. Among them, 66 (2.5%) declined to consent and were screened out, and 300 (11.5%) who began the survey were excluded from analysis because they did not complete it. A further 87 (3.3%) completed the survey but were excluded because either their completion time was shorter than 120 seconds or they did not pass the platform’s AI concentration test. The responses of the remaining 2153 participants were eligible for analysis. Feedback on the BWS survey is shown in Multimedia Appendix 2.

### Participants’ Characteristics

Table 2 presents the characteristics of the study participants. Nearly 55% (1192/2153) were female, and the mean age was 31.4 years. Most participants (1765/2153, 81.9%) had completed tertiary or higher education, and approximately 70% (1523/2153) were urban residents. Additionally, 63.5% (1368/2153) of participants had been infected with COVID-19 in the past year. Almost 90% (1921/2153) reported experiencing COVID-19–related depression to some extent, and 68.6% (1478/2153) usually used smartphone apps to manage their health.

**Table 2:** Respondents’ background characteristics (n=2153).

Characteristics		Values
**Sex, n (%)**		
	Male	962 (44.7)
	Female	1192 (55.3)
**Educational attainment, n (%)**		
	Secondary or below	389 (18.1)
	Tertiary or above	1765 (81.9)
**Employment, n (%)**		
	Full-time	1785 (82.9)
	Unemployed	63 (2.9)
	Retired	24 (1.1)
	Student	282 (13.1)
**Location of residence, n (%)**		
	Rural resident	631 (29.3)
	Urban resident	1523 (70.7)
**Self-perceived income, n (%)**		
	Lower than average	441 (20.5)
	Equal to average	1430 (66.4)
	Higher than average	283 (13.1)
**Health status this week, n (%)**		
	Not well	155 (7.2)
	Average	1241 (57.6)
	Very well	758 (35.2)
**History of infection with COVID-19, n (%)**		
	Yes	1368 (63.5)
	No	786 (36.5)
**Experience of COVID-19–related depression, n (%)**		
	None	233 (10.8)
	Sometimes	1344 (62.4)
	Often	476 (22.1)
	Always	101 (4.7)
**Use of apps to manage health, n (%)**		
	Yes	1478 (68.6)
	No	676 (31.4)
Age (years), mean (SD)		31.4 (8.5)
Age (years), range		18-72

### Outcomes of BWS Analysis

Table 3 presents the distribution of BWS scores for each attribute. The BWS score range for each attribute was between –5 and 5, with the highest proportion of responses centered around 0.

Count analysis demonstrated that participants rated “surveillance and monitoring of infected cases” as the most important app function (mean BWS score 0.98, 95% CI 0.88-1.07). “Mental health therapy” was selected as the least important function, with a mean BWS score of −1.12 (95% CI −1.2 to −1.04; Table 4).

Both the CLM and MXL models exhibited similar estimations (Table 4). Each attribute’s importance was measured relative to “mental health therapy,” which was consistently rated as the least important attribute (ie, this was used as the reference). Both models showed that “surveillance and monitoring of infected cases” (odds ratio [OR] 2.73, 95% CI 2.620-2.850 for CLM, mean coefficient 1.52 for MXL) was the function most favored by the participants, followed by “quick self-screening” (OR 2.47, 95% CI 2.620-2.850 for CLM, mean coefficient 1.39 for MXL). The remaining attributes were of intermediate importance, except for “platform for experience sharing,” which was close to the reference attribute (“mental health therapy”) in the CLM. Additionally, the MXL model provided the SD estimation for each attribute. The significant results indicated that there was substantial heterogeneity regarding the relative importance for all items.

**Table 3:** Distribution of mean best-worst scaling scores for each function of the mobile phone app (n=2153).

Function	Best-worst scaling score, n (%)										
	–5	–4	–3	–2	–1	0	1	2	3	4	5
Surveillance and monitoring of infected cases	28 (1.3)	32 (1.5)	71 (3.3)	127 (5.9)	267 (12.4)	406 (1.8)	364 (16.9)	307 (14.3)	248 (11.5)	130 (6)	174 (8.1)
Quick self-screening	11 (0.5)	49 (2.3)	64 (3)	152 (7.1)	299 (13.9)	468 (21.7)	361 (16.8)	292 (13.6)	208 (9.7)	114 (5.3)	136 (6.3)
Early detection of infected cases	9 (0.4)	15 (0.7)	63 (2.9)	146 (6.8)	254 (11.8)	545 (25.3)	459 (21.3)	316 (14.7)	193 (9)	96 (4.5)	58 (2.7)
Informing prevention protocol	1 (0)	16 (0.7)	47 (2.2)	160 (7.4)	335 (15.6)	493 (22.9)	470 (21.8)	336 (15.6)	171 (7.9)	76 (3.5)	49 (2.3)
Contact tracing	32 (1.5)	49 (2.3)	106 (4.9)	194 (9)	344 (16)	454 (21.1)	393 (18.2)	278 (12.9)	199 (9.2)	71 (3.3)	34 (1.6)
Mobile-based consultation for treatment	25 (1.2)	88 (4.1)	145 (6.7)	229 (10.6)	342 (15.9)	440 (20.4)	323 (15)	263 (12.2)	172 (8)	112 (5.2)	15 (0.7)
Mobile-based consultation for rehabilitation	11 (0.5)	54 (2.5)	118 (5.5)	277 (12.9)	443 (20.6)	530 (24.6)	366 (17)	206 (9.6)	102 (4.7)	27 (1.3)	20 (0.9)
Offering education	67 (3.1)	119 (5.5)	181 (8.4)	337 (15.6)	416 (19.3)	459 (21.3)	291 (13.5)	161 (7.5)	69 (3.2)	31 (1.4)	23 (1.1)
Supporting medical research	73 (3.4)	124 (5.8)	213 (9.4)	275 (12.8)	397 (18.4)	521 (24.2)	282 (13.1)	135 (6.3)	76 (3.5)	45 (2.1)	13 (0.6)
Platform for experience sharing	122 (5.7)	127 (5.9)	198 (9.2)	318 (14.8)	374 (17.4)	501 (23.3)	283 (13.1)	126 (5.8)	72 (3.3)	23 (1.1)	10 (0.5)
Mental health therapy	19 (0.9)	204 (9.5)	319 (14.8)	370 (17.2)	417 (19.4)	431 (20)	251 (11.7)	90 (4.2)	36 (1.7)	15 (0.7)	2 (0.1)

**Table 4:** Results of the BWS^a^ analysis.

	BWS score	Mean BWS score (95% CI)	Conditional logit model^b^				Mixed logit model^c^		
			Coefficient^d^	SE	Odds ratio (95% CI)	Mean coefficient^d^		SE	SD^d^
Surveillance and monitoring of infected cases	2110	0.98 (0.88 to 1.07)	1.005	0.021	2.733 (2.620 to 2.850)	1.52		0.036	1.321
Quick self-screening	1659	0.77 (0.68 to 0.86)	0.905	0.021	2.472 (2.370 to 2.577)	1.396		0.036	1.216
Early detection of infected cases	1504	0.69 (0.62 to 0.77)	0.869	0.021	2.385 (2.287 to 2.487)	1.38		0.041	0.8
Informing prevention protocol	1339	0.62 (0.55 to 0.69)	0.832	0.021	2.299 (2.205 to 2.397)	1.329		0.036	0.489
Contact tracing	594	0.28 (0.19 to 0.36)	0.671	0.021	1.956 (1.876 to 2.039)	1.114		0.032	0.97
Mobile-based consultation for treatment	176	0.08 (0.01 to 0.17)	0.579	0.021	1.784 (1.711 to 1.860)	1.055		0.032	0.893
Mobile-based consultation for rehabilitation	–330	–0.15 (–0.23 to –0.08)	0.461	0.021	1.585 (1.521 to 1.625)	0.867		0.031	0.254
Offering education	–1385	–0.64 (–0.73 to –0.56)	0.229	0.021	1.258 (1.207 to 1.311)	0.575		0.037	0.78
Supporting medical research	–1422	–0.66 (–0.75 to –0.57)	0.218	0.021	1.244 (1.193 to 1.296)	0.544		0.041	0.687
Platform for experience sharing	–1829	–0.85 (–0.94 to –0.76)	0.127	0.021	1.135 (1.089 to 1.183)	0.357		0.046	0.901
Mental health therapy	–2416	–1.12 (–1.2 to –1.04)	Reference level	N/A^e^	N/A	Reference level		N/A	N/A

^a^BWS: best-worst scaling.

^b^Akaike information criterion=136594.3; log likelihood=–70980.9.

^c^Akaike information criterion=129642.2; log likelihood=–64780.

^d^*P*<.001 for all values in column.

^e^N/A: not applicable.

### Stratification Analysis

Stratified analyses showed significant differences across subgroups (Table 5). Compared to individuals who had never been infected with COVID-19, those who had been infected indicated that “mobile-based consultation for treatment” and “mobile-based consultation for rehabilitation” were more important. On the other hand, “offering education” was seen as more attractive by those who had not been infected with COVID-19. Rural residents preferred functions such as “offering education,” “supporting medical research,” and “mental health therapy” more than their urban counterparts.

**Figure 1:**
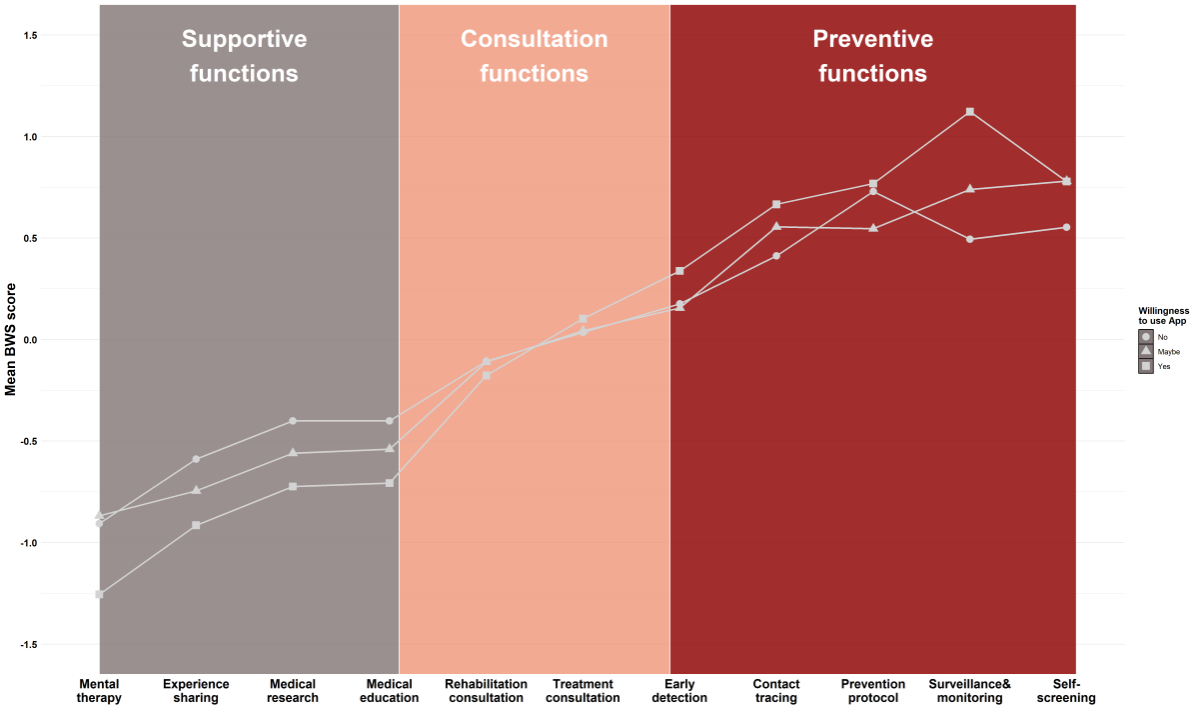
Mean BWS score for attributes based on respondents’ willingness to use the smartphone app. BWS: best-worst scaling.

Mean BWS scores for the participants’ willingness to use this smartphone app (yes, no, or maybe) are shown in Figure 1. All functions can be regrouped into 3 domains. Participants who preferred to use the app were more likely to assign a high weight to the preventive functions (eg, contact tracing) than those who did not prefer to use it. Conversely, participants who showed lower willingness to use the app tended to indicate a higher preference for supportive functions than those who preferred to use it.

**Table 5:** Stratified analysis of best-worst scaling scores by respondents’ COVID-19–related characteristics and sociodemographic characteristics.

	COVID-19 infected vs noninfected	*P* value	COVID-19–related depression vs no depression	*P* value	Use of apps to manage health vs no use	*P* value	Men vs women	*P* value	Rural residents vs urban residents	*P* value	≤ Secondary education vs ≥ tertiary education	*P* value
	Δ^a^ (effect size)		Δ (effect size)		Δ (effect size)		Δ (effect size)		Δ (effect size)		Δ (effect size)	
Surveillance and monitoring of infected cases	0.05 (0.01)	.89	0.06 (0.05)	.34	0.4 (0.19)	.002	–0.15 (0.03)	.31	–0.06 (0.08)	.02	–0.02 (0.03)	.69
Quick self-screening	0.1 (0.04)	.21	–0.6 (0.04)	.69	0.31 (0.09)	.07	–0.16 (0.03)	.35	–0.16 (0.1)	.002	–0.54 (0.19)	≤.001
Early detection of infected cases	–0.11 (0.06)	.11	–0.09 (0.03)	.28	–0.07 (0.04)	.79	0.24 (0.08)	.02	–0.21 (0.13)	.001	0.04 (0.04)	.58
Informing prevention protocol	–0.05 (0.02)	.74	0.06 (0.07)	.25	0.05 (0.04)	.99	0.02 (0.01)	.71	–0.04 (0.03)	.80	0.23 (0.09)	.16
Contact tracing	–0.09 (0.13)	.31	–0.08 (0.04)	.53	0.07 (0.04)	.76	–0.22 (0.08)	.02	–0.16 (0.05)	.67	–0.01 (0.05)	.16
Mobile-based consultation for treatment	0.21 (0.07)	.04	–0.13 (0.06)	.40	0.41 (0.12)	.03	–0.07 (0.02)	.54	–0.1 (0.12)	.002	–0.47 (0.17)	≤.001
Mobile-based consultation for rehabilitation	0.18 (0.07)	.02	0.06 (0.03)	.65	–0.08 (0.07)	.16	0.13 (0.05)	.11	0.16 (0.05)	.61	0.02 (0.03)	.45
Offering education	–0.24 (0.09)	.01	–0.02 (0.09)	.07	0.43 (0.18)	.002	0.27 (0.06)	.04	0.07 (0.17)	≤.001	0.25 (0.1)	.05
Supporting medical research	0.01 (0.02)	.74	–0.19 (0.09)	.15	0.3 (0.15)	.04	–0.01 (0.02)	.52	0.32 (0.18)	≤.001	0.45 (0.19)	≤.001
Platform for experience sharing	0.05 (0.02)	.73	0.08 (0.04)	.49	–0.01 (0.05)	.83	–0.04 (0.02)	.50	0.1 (0.06)	.56	–0.22 (0.08)	.04
Mental health therapy	0.01 (0.01)	.76	0.51 (0.2)	≤.001	0.37 (0.15)	.003	–0.01 (0.01)	.92	0.07 (0.11)	≤.001	0.25 (0.1)	.05

^a^Δ indicates the difference in means.

## Discussion

### Principal Findings

This is the first study to use the BWS method to inform policymakers about the importance of various smartphone app functions based on the public’s preferences for dealing with health risks and maintaining a good quality of life during the pandemic. We found surveillance- and prevention-related functions were identified as more crucial than others to uncover the meaning of the relative importance respondents attach to smartphone apps; this information can be used to inform the development of such apps to prepare for the outbreak of future public health crises. Although the value of smartphone apps in generating population-wide, real-time, and highly informative data in fighting the COVID-19 pandemic has been widely recognized, it is important to acknowledge that there are limitations to the use of smartphone apps in managing a pandemic. Due to limited resources and technical constraints, all necessary functions cannot be provided by a single app. A trade-off must be made to ensure that the most demanding functions can be included. Our findings provide quantitative evidence on which functions should be given higher priority than others based on the perspective of the general population in China.

Public health surveillance measures were listed as the most important functions of the app in our study, which is not a surprise. This is in line with previous findings that governments, private firms, and universities worldwide have used smartphone apps to surveil infected cases as a potential tool to manage the COVID-19 pandemic [20]. In this study, we identified that surveillance and monitoring of infected cases was the most preferred app function of respondents and was approximately 10.5% and 14.6% more important than the second-ranked “quick self-screening” and third-ranked “early detection of infected cases” functions, respectively. This finding is interesting because it shows that people in China may prioritize containing infected individuals over rapid detection during the pandemic. This preference reflects people’s belief that this approach can result in a strong and effective response to the outbreak of COVID-19 and could be useful in handling future public health crises as a top priority in promoting public health and safety. This aligns with the Chinese government’s COVID-19 policies and strategies, which focus on controlling the spread of the virus through measures such as mass and instant lockdowns, compulsory travel restrictions, and strict contact tracing [21,22]. However, some critics argue that this approach may come at the cost of individual liberties and privacy. Future studies about a balance to be found between public health and personal freedoms should be encouraged.

It was unexpected that providing mental health therapy and services via smartphone apps was consistently selected as the least important function in this study. It is in contrast to previous studies, which reported that mental health services are useful to some extent. For example, Liu et al [23] indicated that web-based mental health services for the COVID-19 epidemic facilitated the development of emergency public interventions and improved the quality and effectiveness of emergency interventions [24]. There may be several reasons for this finding. First, problematic use of smartphone apps can result in issues and a decrease in the desire to continue using them. For instance, a recent systematic review indicated that there are numerous digital mental health tools available in China, but a lack of education about their proper use has a negative impact on individuals’ mental health and emotional well-being [25]. Another reason might be concerns about safety and security of personal information using smartphone apps. People may not seek web-based mental health therapy due to worry that their data could be compromised or that their privacy could be violated [26]. Additionally, some people may have difficulty navigating web-based platforms or may lack access to the necessary technology to participate in web-based mental health services due to low eHealth literacy [27]. With the pandemic, it is more important than ever to take care of one’s mental health. Given the government has invested a significant amount of resources into web-based mental health services, it is essential to encourage people to use these services.

Although the Chinese government has encouraged and invested in telemedicine for years, our study found that the treatment-related functions of mobile apps are not considered very important. This indicates that previous concerns, such as lack of reliable access to the internet [28], privacy and security concerns [29], and difficulty building rapport with health care providers via web or mobile-based communication [30], have not been adequately addressed. Additionally, we found respondents showed a lack of interest in sharing their experiences in fighting the pandemic or supporting medical research. However, sharing experiences and uploading data can provide valuable insights into the ways that the virus can affect individuals, which can help researchers and health care professionals better understand and manage the disease. We suggest conducting further research to encourage the sharing of experiences. This can help reduce stigma and the spread of misinformation, as well as promote a more supportive and informed community response to the pandemic.

Further stratified analysis confirmed that there was heterogeneity of preference in different population groups. For example, individuals who had not been infected by COVID-19 were more likely to rate “offering medical education” as more important than those who had been infected. However, individuals who had been infected rated mobile-based treatment or rehabilitation services as more important. These findings reflect changes in the public’s needs and preferences during the pandemic and provide suggestions for the development of different app functions for managing health, whether the users are patients or healthy individuals. Additionally, we found that preferences for mobile app features in urban and rural residents were quite different. For instance, the rural population rated providing mental health services as being more important than did urban residents. This is reasonable, as in China, for various reasons related to culture, socioeconomics, and health care, people with mental health needs have long been underserved [31]. Although treatment for severe mental illness was incorporated into the national public health service in 2009 [32], we found that during the pandemic, the provision of services may not have been sufficient, particularly for rural populations. Therefore, it is important to further study the potential of telemedicine to improve mental health services.

The BWS case 1 method is a conventional ranking method that provides a simple rank ordering of scores to potential factors. It offers several benefits, such as reduced cognitive burden, easier choice tasks, smaller sample sizes, full rankings instead of partial rankings, and reducing personal response–style bias. However, BWS case 2 and case 3 approaches are increasingly identified as promising new ways to generate richer preference information than the case 1 method. Therefore, the use of these alternative designs should be encouraged in future studies.

Personal preference has been identified as a key mechanism for understanding users’ app adoption behavior. This emphasizes the value that users can gain from using apps and proposes that when users’ personal needs are met, they are more likely to continue using apps [33]. Our findings can facilitate the creation of effective strategies to develop mobile apps that fully consider users’ preferences and willingness to manage their health, not only during a pandemic, but also during other public health crises.

### Limitations

Several limitations should be addressed. First, all data used in this study were obtained from a web-based survey. This method of data collection may have introduced selection bias because individuals who are not familiar with web-based surveys may have been excluded, even though respondents in this study came from various regions of China. Second, to reduce the cognitive burden on respondents, we included only 11 smartphone app functions in the BWS questionnaire and left out other possible functions and services. This limitation may affect the generalizability of our findings. Last, since this study used a cross-sectional design, individuals’ long-term state of health and the associated changes were not taken into account in the model estimation. This may affect the validity of our findings.

### Conclusion

This study used a BWS method to rank the importance of features of smartphone apps that provide health care services during pandemics based on general population preferences in China. The 3 most vital functions according to the respondents were “surveillance and monitoring of infected cases,” “quick self-screening,” and “early detection of infected cases.” In contrast, “mental health therapy” was listed as the least vital function for managing health during the pandemic. Our findings provide empirical evidence for decision-makers to develop eHealth policies and strategies that address future public health crises from a person-centered care perspective. Continued use of apps and smart investment in digital health can help improve health outcomes and reduce the burden of disease on individuals and communities.

## Appendix

Multimedia Appendix 1 Explanations of each best-worst scaling attribute.

Multimedia Appendix 2 Feedback on best-worst scaling survey.

## Abbreviations

AIC: Akaike information criterion
BWS: best-worst scaling
BIBD: balanced incomplete block design
CLM: conditional logit model
LL: log likelihood
MXL: mixed logit model
OR: odds ratio

## Data Availability

The data sets generated during and/or analyzed during this study are not publicly available but are available from the corresponding author on reasonable request.
