# Supplementary material for: Investigating Individuals’ Preferences in Determining the Functions of Smartphone Apps for Fighting Pandemics: Best-Worst Scaling Survey Study
Source: J Med Internet Res. 2023 Aug 15;25:e48308. doi: 10.2196/48308 (PMC10466146; doi:10.2196/48308)
Supplement: Multimedia Appendix 1 [file jmir_v25i1e48308_app1.docx]

Table 1 Details of each function

| Functions | Details |
| --- | --- |
| Surveillance and monitoring of infected cases | Provide robust data for monitoring the spread and intensity of respiratory virus activity and circulating variant viruses. |
| Quickly self-screening | Text or videos to show how to use self-screening tools and guidelines of confirmed infection |
| Early detection of infected cases | Self-reported system to record infected cases |
| Informing prevention protocol | Providing protocol to inform people how to protect themselves avoiding to be infected |
| Contact tracing | Self-reported data about his/her trace after infection |
| Mobile-based consultation for treatment | Medical professionals providing treatment suggestions via a web-based platform |
| Mobile-based consultation for rehabilitation | Medical professionals providing rehabilitation suggestions via a web-based platform |
| Offering education | Providing materials to understand the virus, vaccine, symptoms and side-effects |
| Supporting medical research | Providing data and informed consent to facilitate research |
| Platform for experience sharing | Providing a platform for people to share their experience and tory in fighting virus |
| Mental health therapy | Providing mental health services, including consultation and treatment via web-based platform |
