# Supplementary material for: Investigating Individuals’ Preferences in Determining the Functions of Smartphone Apps for Fighting Pandemics: Best-Worst Scaling Survey Study
Source: J Med Internet Res. 2023 Aug 15;25:e48308. doi: 10.2196/48308 (PMC10466146; doi:10.2196/48308)
Supplement: Multimedia Appendix 2 [file jmir_v25i1e48308_app2.docx]

Table A1 Feedback on BWS survey

| **Item** | **Option** | **%** |
| --- | --- | --- |
| Was the presentation of health states clear? | Very clear | 28.3 |
|  | Clear | 46.9 |
|  | Neither clear nor unclear | 21.2 |
|  | Unclear | 3.1 |
|  | Very unclear | 0.6 |
| How difficult it was to choose between pairs of health states? | Very difficult | 2.9 |
|  | Difficult | 21.4 |
|  | Neither easy nor difficult | 32.5 |
|  | Easy | 32.2 |
|  | Very easy | 21.1 |
